# Effectiveness of digital tools for smoking cessation in Asian countries: a systematic review

**DOI:** 10.1080/07853890.2023.2271942

**Published:** 2024-02-12

**Authors:** Khang Wen Goh, Long Chiau Ming, Yaser Mohammed Al-Worafi, Ching Siang Tan, Andi Hermansyah, Inayat Ur Rehman, Zahid Ali

**Affiliations:** a Faculty of Data Science and Information Technology, INTI International University, Nilai, Malaysia; b Department of Pharmacy Practice, Faculty of Pharmacy, Universitas Airlangga, Surabaya, Indonesia; c PAPRSB Institute of Health Sciences, Universiti Brunei Darussalam, Gadong, Brunei Darussalam; d School of Medical and Life Sciences, Sunway University, Sunway City, Malaysia; e College of Medical Sciences, Azal University for Human Development, Sana’a, Yemen; f College of Pharmacy, University of Science and Technology of Fujairah, Fujairah, UAE; g School of Pharmacy, KPJ Healthcare University, Nilai, Malaysia; h Department of Pharmacy, Garden Campus, Abdul Wali Khan University Mardan, Mardan, Pakistan; i Department of Pharmacy, University of Peshawar, Peshawar, Pakistan

**Keywords:** Tobacco use, Cognitive behavioural theory, Human and health, Tobacco control, Tobacco addiction, Public health, Medicine

## Abstract

**Aim:**

The use of tobacco is responsible for many preventable diseases and deaths worldwide. Digital interventions have greatly improved patient health and clinical care and have proven to be effective for quitting smoking in the general population due to their flexibility and potential for personalization. However, there is limited evidence on the effectiveness of digital interventions for smoking cessation in Asian countries.

**Methods:**

Three major databases – Web of Science (WOS), Scopus, and PubMed – for relevant studies published between 1 January 2010 and 12 February 2023 were searched for studies evaluating the effectiveness of digital intervention for smoking cessation in Asian countries.

**Results:**

A total of 25 studies of varying designs were eligible for this study collectively involving a total of *n* = 22,005 participants from 9 countries. Among different digital tools for smoking cessation, the highest abstinence rate (70%) was reported with cognitive behavioural theory (CBT)-based smoking cessation intervention *via* Facebook followed by smartphone app (60%), WhatsApp (59.9%), and Pharmacist counselling with Quit US smartphone app (58.4%). However, WhatsApp was preferred over Facebook intervention due to lower rates of relapse. WeChat was responsible for 15.6% and 41.8% 7-day point prevalence abstinence. For telephone/text messaging abstinence rate ranged from 8-44.3% and quit rates from 6.3% to 16.8%. Whereas, no significant impact of media/multimedia messages and web-based learning on smoking cessation was observed in this study.

**Conclusion:**

Based on the study findings the use of digital tools can be considered an alternative and cost-effective smoking cessation intervention as compared to traditional smoking cessation interventions.

## Introduction

Tobacco use is a significant cause of morbidity, mortality, and impoverishment globally, which can be prevented [1]. There are many hazards of cigarette smoking, both to the smoker and to those around them. Each year, tobacco claims the lives of over 8 million individuals, with more than 7 million deaths attributed to direct tobacco use, while around 1.2 million deaths occur due to non-smokers being exposed to second-hand smoke [2]. It is estimated that if current smoking trends continue, the number of deaths caused by smoking could increase to 8.3 billion by the year 2030 [3].

In the last century, approximately 100 million people died from tobacco use, primarily in developed countries [4,5]. Approximately one billion people may die this century, mostly in low- and middle-income nations, if current tobacco usage persists [1,3,5,6]. Among low-income countries, the dominance and usage of tobacco-related products smoking are increasing to a greater extent [7], and is reported that almost 80% of global tobacco smokers are residing in these low-income countries [2]. In South Asia, about 25.2% of men and 3.26% of women smoke [8], which is a serious threat to public health and is one of the modifiable risk factors contributing to major non-communicable diseases in this region [9]. In the South and Southeast Asian region, the mortality rate is very alarming, and roughly over one million individuals die every year due to tobacco smoking [10]. Smoking has a significant impact on economic costs and health-related expenditures due to smoking-attributable diseases exerting a huge economic burden worldwide in technologically advanced countries [11]. In order to cope with the World Health Organization’s target of minimizing the deaths resulting from cardiovascular diseases, cancer, diabetes, and respiratory diseases in people aged 30–70 years by 25% by the year 2025, one of the strategy is by reducing cigarette consumption that can be one of the best and most cost-effective approach for prevention approach in South Asian countries [1]. The quit rate for smoking reported by previous literature in developed countries showed that self-reported smoking cessation rate was about 19% for 28 days or longer [12], while another study reported the quit rate of 13% *via* the use of mobile apps [13]. Quitting smoking can be a difficult challenge that sometimes requires many attempts before success is achieved, nicotine dependence is a complex disorder [14]. Data privacy and security of apps is also one of the concern by the smoking quitters followed by difficulty in locating effective, operational apps that safeguard user privacy [15]. The online available apps for smoking cessation raised question on their efficacy as well, most apps have not been tested in clinical studies which also act as a challenge to gain the desired outcome when used the smoker to quit smoking [16,17].

Cigarette smoking is a significant public health concern in many Asian countries, with high prevalence rates and associated health risks. The prevalence rates vary between countries and regions, but some general trends can be observed. China is the world’s largest producer and consumer of tobacco, and smoking is prevalent among men, with a prevalence rate of around 52.1%. However, smoking rates among women are lower, at around 2.7% [18]. Indonesia has one of the highest smoking rates in the world, with a prevalence rate of around 63% for men and 5% for women in 2019 [19]. Japan has a relatively high smoking rate, with a prevalence rate of around 38.4% for men and 13% for women during 2001–2016 [20]. South Korea has a high smoking rate among men, with a prevalence rate of around 40–50%. However, smoking rates among women are lower, at around 4–8% [21]. India has a lower smoking prevalence rate compared to other Asian countries, with a prevalence rate of around 17.5% for men and 1.2% for women [22]. The smoking prevalence rate in the Philippines is relatively high, with a prevalence rate of around 40.9% for men and 8.2% for women [23]. The smoking prevalence rate in Vietnam is high, with a prevalence rate of around 45.3% for men and 1.1% for women [24].

As evident from the literature, smoking is a major public health issue that has been linked to numerous health problems, including cancer, heart disease, and respiratory illnesses. However, many people still find it difficult to quit [25], despite the well-known associated risks with smoking. Fortunately, the development of digital tools for tobacco cessation has made it easier for individuals to quit smoking and maintain abstinence [14]. These digital tools can take various forms, including mobile apps, web-based programs, and social media platforms, among others. However, the effectiveness of these digital tools in helping individuals quit smoking is still subject to debate, and more research is needed to determine their efficacy, especially in Asian countries with high prevalence rates and associated health risks.

Previous literature demonstrated that digital tools and apps are effective in smoking cessation in other part of world, the current literature is partial due to lack of precise systematic review reporting the impact of digital tools in smoking cessation among Asian countries. We believe that a systematic analysis exploring the effectiveness of digital tools in smoking cessation among Asian countries will certainly enrich the partially explained gap in knowledge. Therefore this study aimed to determine the existing evidence on the effectiveness of digital tools in helping individuals quit smoking.

## Methods

To conduct this systematic review, the guidelines outlined by the Cochrane Collaboration and the Preferred Reporting Items for Systematic Reviews and Meta-Analyses (PRISMA) statement [26] were followed.

### Study duration

We searched three major databases – Web of Science (WOS), Scopus, and PubMed – for relevant studies published between 1 January 2010 and 12 February 2023.

### Search strategies

The strategic search terms used medical subject headings (MeSH) and keywords, and the following text terms were combined with Boolean operators: (digital tools OR digital interventions OR eHealth OR mHealth OR smartphone apps OR text messaging OR social media OR web-based interventions OR online interventions OR internet OR Telemedicine OR Mobile Applications OR Health Education OR Health Promotion) AND (tobacco cessation OR smoking cessation OR nicotine dependence OR quit smoking OR quit tobacco OR nicotine replacement therapy OR NRT OR Smoking Cessation OR Tobacco Use Cessation OR Nicotine Replacement Therapy OR Varenicline OR Bupropion OR Counselling OR e-cigarette OR vape).

## Study selection

### Inclusion Criteria

Original research articles having an observational and experimental design, published in peer-reviewed English journals published between the ranges 1 January 2010 till 12 February 2023, used smoking cessation interventions delivered *via* a digital method, and studies conducted in Asian countries were included.

### Exclusion criteria

All systematic reviews, meta-analyses, review articles, case reports, advertisements, thesis, opinions, letters to the editor, conference proceedings, and qualitative studies were excluded.

### Data extraction

All selected articles were extracted independently by IUR and ZA by using a standardized extraction form designed for this systematic review. The following information was extracted from individual articles: author’s name, publication year, type of study design, year of research study conducted, country name of the study conducted, respondent’s information, sample size, type of digital or mobile-related intervention used, and intervention duration at baseline and endpoint information, statistical analysis used and outcomes measurement at baseline and endpoint i.e. Point Prevalence Abstinence (PPA) in 24 h, 7 days up to months as a designated endpoint by the studies. Disagreement on evaluation for inclusion was resolved by discussion of the authors, and if necessary, a third reviewer (LCM) was included in the discussion to reach a consensus.

### Data analysis

As the studies that meet the inclusion criteria were of diversified nature and were not combinable for meta-analysis, keeping in view the nature of the data extracted the data were shortlisted for qualitative synthesis instead of quantitative synthesis.

## Results

### Study selection

**Figure 1.**
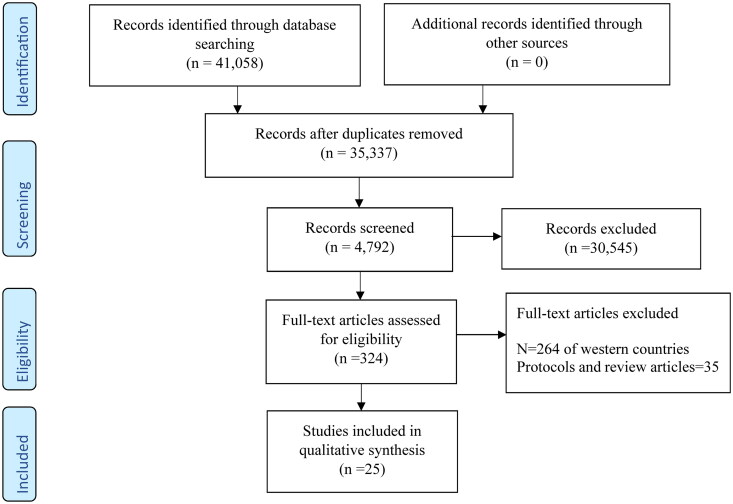
PRISMA flow diagram of study selection.

A total of *n* = 41,058 related research articles were identified, having *n* = 434 from PubMed, *n* = 13,974 from Web of Science (WOS), and *n* = 26,650 from Scopus. After the removal of duplicates *n* = 5721 the articles obtained were *n* = 35,337. After applying the filters i.e. English language, human species, and studies published between 1 January 2010 and 12 February 2023; *n* = 4792 articles were obtained. By reviewing and screening the title and abstract of the articles *n* = 324 were obtained and upon further applying the filter (studies of Asian countries) a total of *n* = 25 studies were included in this systematic review as shown in Figure 1.

### Characteristics of selected studies

Of the included 25 studies, *n* = 9 were from China [2,27–34]; *n* = 3 were from Hong Kong [10,35,36]; *n* = 3 were from Turkey [37–39]; *n* = 3 from Thailand [40–42]; *n* = 2 were from Taiwan [43,44]; *n* = 2 from Japan [45,46]; *n* = 1 from South Korea [47]; *n* = 1 from Indonesia [48]; *n* = 1 from Jordon [23] as shown in Table 1.

**Table 1.** Characteristics of included studies.

Author	Year	Country	Intervention Type	Control Group	Intervention to the Experimental group	Duration	Study Design	Total Subjects	Control	Experimental
SMS										
Ybarra et al. [37]	2012	Turkey	Text messaging	Control arm received a brochure that provided similar information about smoking cessation	The text messaging intervention, 6 weeks of daily messages aimed at giving participants skills to help them quit smoking	3 months	Randomized control trial	151	75	76
White et al. [42]	2013	Thailand	Counseling and SMS	Smoking-cessationcounseling	Counseling and text message reminders	6 months	Randomized control trial	215	69	132
Lin et al. [33]	2023	China	Text message for smoking cessation	A non-personalized text message that encouraged, practical advice to help maintain cessation and health effects of smoking	Text message for smoking cessation having personalized messages i.e. 1 to 2 text messages per day for 3 months through the app	6 months	Randomized control trial	722	362	360
Telephone, SMS										
Guo et al. [44]	2014	Taiwan	Telephone, SMS	NA	Education, Acupressure, Telephone, SMS	4 month	Pre-post study	143	65	78
Chan et al. [10]	2015	Hong Kong	Telephone, SMS	No SMS and Telephone	Telephone call with 5-min nurse-led telephone counseling within seven days after enrolment 8 text messages	12 months	Randomized control trial	1003	330	Tel= 338 SMS= 335
Social Media										
Cheung et al. [35]	2015	Hong Kong	WhatsApp and Facebook	Smoking cessation self-help booklet	Online group discussion for 2 months by a trained smoking cessation counselor either WhatsApp or Facebook along with a smoking cessation self-help booklet	6 months	Randomized control trial	WhatsApp 42 Facebook 40	54	WhatsApp= 42 Facebook= 40
Durmaz et al. [39]	2019	Turkey	WhatsApp application	Usual care by physicians trained on quitting tobacco by giving either a motivational interview or a quitting counseling	WhatsApp application added to the usual care (Usual care by physicians trained on quitting tobacco by giving either a motivational interview or quitting counseling)	6 months	Randomized control trial	132	88	44
Luo et al. [31]	2022	China	WeChat-“Quit Smoking Help"	No SMS	Intervention group 1 for 2 weeks received a total of 20 smoking-related while intervention group 2 similarly for 2 weeks received 20 smoking-related messages along with an additional week of additional messages for oral health	4 weeks	Randomized control trial	403	132	271
wang et al. [34]	2019	China	Chat-based instant messaging support	Face-to-face smoking cessation by smoking cessation ambassadors	Face-to-face smoking cessation by smoking cessation ambassadors and additionally Chat-based cessation support *via* WhatsApp messages	6 months	Randomized control trial	1185	594	591
Luk et al. [36]	2023	Hong Kong	Personalized chat messages *via* WhatsApp on relapse prevention	Standard treatment for smoking cessation (behavioral support, NRT, bupropion, and varenicline)	Standard treatment for smoking cessation (behavioral support, NRT, bupropion, and varenicline) with additional chat messaging on *via* WhatsApp for smoking relapse prevention	3 months	Randomized control trial	108	54	54
Chu et al. [32]	2023	China	WeChat and quitline (WQ)	NA	WeChat and quitline (WQ) with a quitline and three WeChat-based cessation services	NA	Prospective cohort study	2221	NA	NA
Telephone										
Huang et al. [2]	2016	China	Telephone counseling intervention	NA	Telephone counseling intervention	3 months	Pre-post study	107	NA	NA
Wu et al. [27]	2016	China	Individual Face-to-face counseling along with telephone counseling	Face-to-face individual counseling	Individual Face-to-face counseling along with telephone counseling	6 month	Non-randomized study	570	117	340
Cheung et al. [28]	2021	China	Telephone	Brief intervention on consuming vegetables and fruit	Received 30-s advice from a physician followed by brief booster advice *via* telephone	12 months	Randomized control trial	10122	6656	3466
Smartphone										
Nomura et al. [45]	2019	Japan	Internet-based video counseling (Telemedicine counseling) with CASC smartphone app	Face-to-face clinic visits with CASC smartphone app	Internet-based video counseling (Telemedicine counseling) with CASC (CureApp Smoking Cessation) smartphone app	6 months	Randomized control trial	115	57	58
Liao et al. [29]	2022	China	CBT-based smoking cessation intervention *via* a smartphone app	NA	CBT-based smoking cessation intervention *via* a smartphone app	33 days	Pre-post study	180	90	90
Chulasai et al. [40]	2022	Thailand	Pharmacist counseling with Quit US smartphone app	Face-to-face and group counseling for smoking cessation counseling by pharmacists Smoking Cessation	Pharmacists’ smoking cessation counseling, by downloading and installing Quit with US app on smartphones	12 weeks	Randomized control trial	273	136	137
Liao et al. [30]	2022	China	Text messaging intervention (‘Happy Quit’)	1 non-interventional message/week	Text messaging intervention (‘Happy Quit’) having a high-frequency messaging (HFM, 3 to 5 messages/ day)and low-frequency messaging (LFM, 3 to 5 messages /week)	6 months	Randomized control trial	1369	411	958
Asayut et al. [41]	2019	Thailand	Pharm Quit: a smartphone app for smoking cessation	Usual smoking cessation services	Pharm Quit: a smartphone app for smoking cessation	6 months	Randomized control trial	156	78	78
Miscellaneous										
Shukr et al. [23]	2023	Jordan	Tobacco smoking-related media messages	NA	Tobacco smoking-related media messages	3 years	Longitudinal study	2174	NA	2174
Choi et al. [47]	2018	South Korea	Web-based e-learning program	NA	Blended learning e-learning program and face-to-face learning program (Web-based e-learning program consisted of 10 courses, each 30-min)	5 weeks	Quasi-experimental design	44	21	23
Koyun et al. [38]	2019	Turkey	Web-based education manual	NA	Web-based educational intervention	6 months	Interventional pre-post study	314	-	314
Wang et al. [43]	2010	Taiwan	Multimedia	Auricular acupressure	Auricular acupressure plus multimedia instruction	10 weeks	Quasi-experimental study	64	32	32
Ismail et al. [48]	2021	Indonesia	Audiovisual Health education on smoking	Health education on the risks of smoking for 15–20 min	Health education intervention using audiovisuals for 4-5 min by videos for different groups i.e. Group 1: Risks of smoking); group 2: Smoking law; group 3: Risk of developing cancer caused by smoking	NA	Quasi-experimental study	152	38	38 × 3

Among the included studies *n* = 9 used telephone/text messaging counseling [2,10,27,28,30,33,37,42,44]; while *n* = 11 used social media/apps smoking cessation interventions [29,31,32,34–36,39–41,45,46]; *n* = 2 used media and multimedia messages [23,43]; *n* = 2 used Web-based e-learning program [38,47] and *n* = 1 used audio-visual health education on smoking [48].

### Study design

Regarding the study design used in the selected articles *n* = 15 were randomized control trials [10,28,30,31,33–37,39–42,45,46]; *n* = 4 were quasi-experimentation/non-randomized control trials [27,43,47,48]; *n* = 4 were pre-post study [2,29,38,44]; *n* = 1 was a prospective cohort study [32] and *n* = 1 was a longitudinal study [23]. These studies also conducted different lengths and types of assessment like 33 days [29]; 4 weeks [31]; 5 weeks [47]; 10 weeks [43]; 12 weeks [40]; 3 months [2,36,37]; 4 months [44]; 6 months [27,30,33,34,38,39,41,42,45]; 12 months [10,28,46] and a longitudinal follow-up for 3 years [23]. One study included four assessments: baseline, at the end of the intervention, and two follow-ups [35].

### Smoking cessation outcomes

Diverse effects were observed across different trials *n* = 11, different social media and mobile applications utilized aiming for smoking cessation interventions [29,31,32,34–36,39–41,45,46]. The success rate of these interventions was particularly noteworthy, with a greater success rate of 70% for participants with smoking cessation interventions based on Facebook, followed by 59.5% of participants with smoking cessation interventions based on WhatsApp [35]. A smartphone app-based smoking cessation strategy that was delivered using a Cognitive Behavioural Theory (CBT) also made it easier for 60% of participants to stop smoking [29]. Furthermore, the 7-day PPA rates with WeChat with quit line were 41.8% [32], and the smoking abstinence rate was 58.4% when using pharmacist counseling with the Quit US smartphone app [40]. While using WeChat-’Quit Smoking Help’ the 28 and 7-day PPA rates were 15.6% in group 1 (which got 20 messages related to smoking for two weeks) and 20.6% in group 2 (which received 20 messages related to smoking for two weeks plus an additional six messages related to dental health for a third week) [31]. Similarly, the abstinence rate in the first month was 65.9% by the use of the WhatsApp app added to the usual care [39], details are shown in Table 1.

Among the studies that used telephone/text messaging counseling [2,10,27,28,30,33,37,42,44] regarding smoking cessation with the use of telephone/text message the findings revealed that at 7-day abstinence was 9.1% and at 12-month follow-up was 8.0% [28]; while the quit rate was 6.3% with text messaging intervention (‘Happy Quit’) and 6.9% respectively [2,30,33]; however a superior continuous abstinence rate of 16.8% was reported while using face-to-face individual counselling plus follow-up telephone counselling [27] followed by 11% individuals quit smoking by using text messaging [37]. However, no significant difference was found by using SMS/telephone intervention in one of the study in which at 12-month follow-up [10]. Another study reported the effectiveness of counselling and SMS with the abstinence rate was 44.3% at the end of the study [42].

Regarding smoking cessation using web base learning studies [38,47] no remarkable impact on smoking cessation was observed. Similarly, the use of media/multimedia messages [23,43] for smoking cessation also showed no significant improvement in smoking cessation among the participants. Regarding audio-visual health education on smoking revealed that the provision of health education using audio-visuals was more effective in increasing the smokers ‘motivation to quit smoking compared to the provision of health education alone [48].

## Discussion

Advancements in technology have enhanced the health and clinical care of patients by utilizing digital interventions. The role of digital interventions on smoking cessation was therefore examined in this study. Social media can provide an acceptable and more viable platform for supporting smoking cessation efforts. This is evident from its capacity to attract and retain smokers online, to administer tailored smoking cessation interventions, and gather meaningful smoking-related results. In addition, it is well understood that digital intervention helps individuals quit smoking by increasing motivation/interest for quitting, sustaining abstinence, and prompting quitting attempts. Although these interventions have been found to be acceptable and possibly effective, more rigorous trials are required to establish their effectiveness, assess the affordability and sustainability of these programs, and determine whether these interventions can be accessed by low-income individuals, young people, or other vulnerable groups who tend to smoke higher than the general population.

In the included studies social media like Facebook [35] and WhatsApp were found to be effective [34–36,39]. Smoking cessation *via* Facebook showed that 70% of individuals in the Facebook intervention group quit smoking [35]. Other studies across the globe also utilized the application of Facebook based smoking cessation among young adults and concluded that this intervention was effective [49]. Similarly, other studies also demonstrated Facebook-based intervention proved to be an innovative and most effective platform for smoking cessation [50,51]. Similarly, the application of WhatsApp was also utilized for smoking cessation and a study by Cheng et al. [35] showed 59.5% of the individuals in the WhatsApp group quit smoking; furthermore, the recent quitters in the WhatsApp intervention group had a slightly lower relapse rate than those in the Facebook intervention group. Another study by Durmaz et al. [39] and Luk et al. [36] also reported that sixth month the abstinence rate among the intervention group with WhatsApp intervention was 40.9% and 31.4% respectively.

Furthermore, the use of smartphone apps along with WeChat [31,32], Pharmacist counseling with Quit US smartphone app [40], and the use of Cognitive behavioral theory (CBT)-based smoking cessation intervention *via* a smartphone app [29] are also tested for smoking cessation among smokers are showed be effective in smoking cessation. Studies by Luo et al. [31] and Chu et al. [32] showed that WeChat intervention on smoking cessation showed that at 7-day PPA rates were 15.6% in group 1 (that received 20 smoking-related messages for 2 weeks) with 20.6% in group 2 (received 20 smoking-related messages for 2 weeks and an extra 6 oral health-related messages for an additional week) and 41.8% respectively. Findings of a meta-analysis revealed that Compared with traditional smoking cessation interventions, digital tools based on the WeChat platform significantly increased the prevalence of abstinence from smoking [52]. WeChat is the most frequently used social media platform among Chinese people globally and the participants reported that the messages enhanced their motivation to quit, offered encouragement, and made them more informed about how to quit [53]. Our included studies showed that face-to-face counseling along with installing Quit with US on their smartphones showed a Smoking abstinence rate of 58.4% [40] and Cognitive behavioral theory (CBT)-based smoking cessation intervention *via* a smartphone app showed 60% of the participants were able to stop smoking from the quit date to the end of the program [29]. A digitally associated interface provides the CBT therapist with the added ability to help the smoker by providing personalized attention to those who want to quit smoking. A digitally clinician-assisted CBT intervention combining pharmacotherapy and behavioural treatment for smoking cessation proved to be an effective approach to achieving smoking cessation [54]. Mobile instant messaging apps (e.g. WhatsApp, Facebook Messenger, and WeChat) are considered to be an alternative for smoking cessation as compared to traditional smoking cessation services due to its widely usage and as an inexpensive alternative as compared to SMS for interactive messaging [34].

Telephone/text messaging interventions are effective tools in smoking cessation, providing support, encouragement, and information to individuals trying to quit smoking. This is evident from our study, in which we observed an abstinence rate in the range of 8–44.3% [28,42], and quit rate in the range of 6.3–16.8% *via* telephone/text messaging intervention [2,27,30,33,37]. These findings are in line with a meta-analysis conducted in Western countries reported 1.37 times more smoking abstinence rate after receiving text message intervention [55]. One study reported no significant difference following SMS/telephone intervention in smoking cessation [10]. However, it’s important to note that quit rates and abstinence rates can vary depending on a variety of factors, such as the type of intervention, the population being studied, and the duration of follow-up. Another study conducted in the United States reported significantly higher PPA in those individuals who received text messages compared to the control group [56]. One study reported no significant difference following SMS/telephone intervention in smoking cessation [10]. However, it’s important to note that quit rates and abstinence rates can vary depending on a variety of factors, such as the type of intervention, the population being studied, and the duration of follow-up.

## Strength and limitations

The strength of this review is that it is the first study that summarizes the effectiveness of digital tools (Facebook, WeChat, WhatsApp, and other smartphone apps) along with telephones/text messages for smoking cessation among active smokers in Asian countries. Another strength of this study was that we can get a deep insight into the most effective digital tool and social apps that helped smokers for 7 days PPA as an effective approach for smoking cessation in Asian countries. The limitation of this study was that a variety of digital tools and telephone/text messages approaches were used along with a combination of other approaches due to which the exact effectiveness of most suitable tool was unable to establish by using a quantitative approach of meta-analysis. Furthermore, due to diverse nature of study designs, heterogeneity and sample sizes were also considered as a limitation of this study and further studies should be designed to address these limitations. One of another possible limitation of this systematic review can be that this study only considered articles published in English language, so possibility exist that findings of articles in other language can be helpful to devising a solution for smoking cessation among Asian countries.

## Conclusion

The included studies in this systematic review suggest that social media platforms such as Facebook and WhatsApp, as well as smartphone apps like WeChat and Quit US, are effective tools for smoking cessation. Facebook-based interventions have shown high quit rates, particularly among young adults. Similarly, WhatsApp interventions have reported comparable abstinence rates but with lower relapse rates compared to Facebook interventions. Smartphone apps based on CBT and pharmacist counselling have also demonstrated high success rates in smoking cessation. The use of mobile instant messaging apps like WhatsApp, Facebook Messenger, and WeChat are considered alternative and inexpensive options for interactive messaging for smoking cessation. Telephone/text messaging interventions have also been shown to be effective in smoking cessation, providing support, encouragement, and information to individuals. However, it is important to note that quit and abstinence rates may vary depending on the type of intervention, the population being studied, and the duration of follow-up. Overall, this study concludes that the use of digital tools can be considered an alternative and cost-effective smoking cessation intervention as compared to traditional smoking cessation interventions. However, it is important to choose the right intervention based on the individual’s needs and preferences.

## Data Availability

Data are not publicly available but may be accessed upon request.
